# Determination of hydrogen site and occupancy in hydrous Mg_2_SiO_4_ spinel by single-crystal neutron diffraction

**DOI:** 10.1107/S2052520618000616

**Published:** 2018-01-25

**Authors:** Narangoo Purevjav, Takuo Okuchi, Xiaoping Wang, Christina Hoffmann, Naotaka Tomioka

**Affiliations:** a Institute for Planetary Materials, Okayama University, Misasa, Tottori 682-0193, Japan; b Neutron Scattering Division, Neutron Sciences Directorate, Oak Ridge National Laboratory, Tennessee TN 37831, USA; c Kochi Institute for Core Sample Research, Japan Agency for Marine-Earth Science and Technology, Nankoku, Kochi 783-8502, Japan

**Keywords:** Earth’s deep mantle, ringwoodite, crystallography of hydrogen, neutron diffraction

## Abstract

Ringwoodite [(Mg,Fe^2+^)_2_SiO_4_ spinel] has been considered as one of the most important host minerals of water in the Earth’s deep mantle. Its extensive hydration was observed in high-pressure synthesis experiments and also by its natural occurrence. Water can dissolve into ringwoodite as structurally bound hydrogen cations by substituting other cations, although the hydrogen site and its occupancy remain unclear. In this study, neutron time-of-flight single-crystal Laue diffraction analysis was carried out for synthetic hydrous ringwoodite. Hydrogen cations were found only in the sites in MgO_6_ octahedra in the ringwoodite structure, which compensated the reduced occupancies of both magnesium and silicon cations. The refined cation occupancies suggest that the most plausible hydration mechanism is that three hydrogen cations simultaneously occupy an MgO_6_ octahedron, whereas four such hydrogenated octahedra surround a vacant SiO_4_ tetrahedron.

## Abstract 2

A single-crystal neutron diffraction study was performed on hydrogen incorporation in ringwoodite, which is the most important host mineral of water in the Earth’s deep mantle. Its hydrogen incorporation mechanism, bonding geometry and occupancy at the relevant hydrogen site were unambiguously revealed.

## 1. Introduction   

Ringwoodite [γ-(Mg,Fe^2+^)_2_SiO_4_] with cubic spinel structure (space group 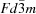) is one of the most abundant mineral phases in the Earth’s mantle (Ringwood & Seabrook, 1962 ▸, Ringwood & Major, 1966 ▸). Based on high-pressure phase equilibrium studies, it was confirmed that ringwoodite is thermodynamically stable at pressures between 17 and 23 GPa at temperatures along the geotherm (Ito & Katsura, 1989 ▸), which corresponds to depths between 520 and 660 km. This depth range is defined as the lower half of the mantle transition zone (MTZ) within the Earth (Dziewonski & Anderson, 1981 ▸; Shearer, 1990 ▸). Subsequently, it was reported that a significant quantity of hydrogen cations could be dissolved into the structure of ringwoodite, which corresponds to at least 2.7 wt% of H_2_O, even if the chemical environment of the crystal growth was not saturated with water (*f*H_2_O < P) (Kohlstedt *et al.*, 1996 ▸). Recently, natural ringwoodite included in a diamond coming from the lower MTZ was discovered for the first time, which contained about 1 wt% of H_2_O within its structure (Pearson *et al.*, 2014 ▸). Based on this data, the amount of water contained in hydrous ringwoodite in the Earth is about the same as the sum of all the oceans (Keppler, 2014 ▸). Thus, hydrous ringwoodite is now considered the main water reservoir in the Earth’s deep mantle.

The crystal structure of hydrous ringwoodite has been studied mostly using X-ray crystallography (Kudoh *et al.*, 2000 ▸; Smyth *et al.*, 2003 ▸). Ringwoodite was reported to maintain its original cubic spinel structure even after hydration and each of its major components (Mg^2+^, Fe^2+^, Si^4+^ and O^2−^) was localized at distinct crystallographic sites. The structure consists of cubic close-packing of oxygen anions (Wyckoff position 2*e*). The cations are located in the centers of *M*O_6_ octahedra (16*d*) and *T*O_4_ tetrahedra (8*a*), which are named *M* and *T* sites, respectively. The magnesium (Mg^2+^) and iron (Fe^2+^) cations are at the *M* sites, while the silicon (Si^4+^) cations are at the *T* sites. It was previously reported that hydrogen cations (H^+^) in Fe-free ringwoodite should substitute by either Mg^2+^ or Si^4+^ cations (Kudoh *et al.*, 2000 ▸). The dominant mechanism was considered to be Mg^2+^ substitution, where the H^+^ was located along the shortest O—O edge of the octahedron (Kudoh *et al.*, 2000 ▸).

However, our recent powder neutron diffraction study on Fe-bearing deuterated ringwoodite with the composition (Mg_1.72_Fe_0.17_D_0.22_)(Si_0.97_D_0.12_)O_4_ revealed that deuterium cations (D^+^) not only substituted Mg^2+^ and Fe^2+^ but also Si^4+^ (Purevjav *et al.*, 2014 ▸). The D^+^ site position and its occupancy were determined quantitatively for the first time for hydrous ringwoodite. Neutron diffraction is isotope specific and highly sensitive to the presence of hydrogen atoms in a crystal structure. Also, neutron diffraction has proven to be a powerful probe for crystallographic analysis of hydrous ringwoodite, as demonstrated by other studies (including our own works) of related minerals in the deep mantle (Sano-Furukawa *et al.*, 2011 ▸; Purevjav *et al.*, 2016 ▸; Tomioka *et al.*, 2016 ▸). Here, we apply single-crystal neutron diffraction to hydrous ringwoodite to resolve the position of its H^+^ site and occupancy using the SNS TOPAZ instrument, a wavelength-resolved time-of-flight (TOF) Laue neutron diffractometer, which employs large-area detector coverage to collect a diffraction pattern surveying continuous and contiguous volumes of three-dimensional reciprocal space. The measured intensity for individual reflections is extracted by integrating well resolved Bragg peaks (Schultz *et al.*, 2014 ▸), which results in much higher sensitivity than powder neutron diffraction. H^+^ has a negative neutron scattering length, in contrast to the positive scattering lengths of Mg^2+^, Si^4+^ and O^2−^. As a result, the existence of tiny amounts of H^+^ amongst many cations with significant positive scattering length densities can be unambiguously revealed from high-resolution single-crystal neutron diffraction. This unique contrast of H^+^ as a negative scatterer provides the most accurate OH^−^ geometry as well as its site occupancy in the parent crystal structure.

## 2. Methodology   

### 2.1. Synthesis and characterization of single crystals   

**Figure 1:**
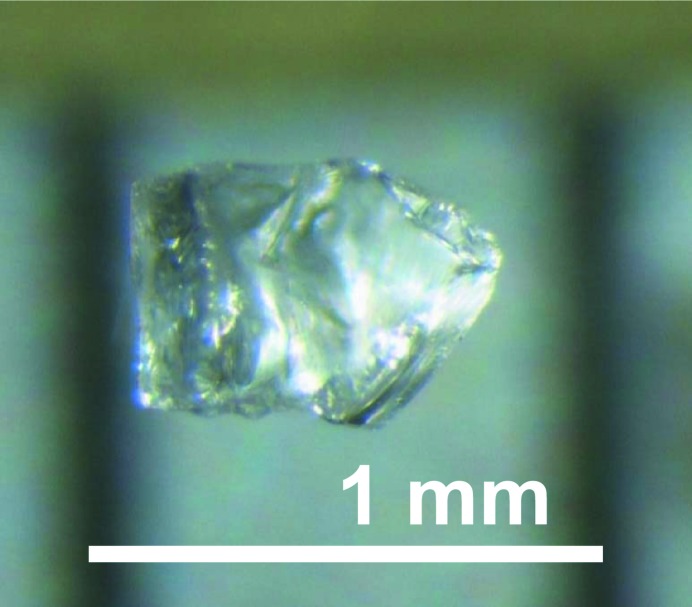
Sample crystal of hydrous ringwoodite used for single-crystal neutron diffraction analysis.

Single crystals of iron-free hydrous ringwoodite were synthesized by using a scaled-up Kawai-type cell, to which we applied our recently established slow-cooling method to grow chemically and physically homogenous crystals of hydrous minerals that exist in Earth’s deep mantle (Okuchi *et al.*, 2015 ▸). A mixture of commercial reagents of MgO, SiO_2_ and Mg(OH)_2_ (1.1:1.0:1.0 by molar ratio) was used as the starting material (Okuchi *et al.*, 2015 ▸). The Kawai-type cell containing about 50 mg of this mixture in a sealed gold capsule (4 mm in outer diameter and 5 mm in length) was compressed to 21 GPa pressure and heated to 1688 K. Then, we slowly decreased the temperature to 1513 K while maintaining the pressure over a period of 10 h. The cell was then quenched to room temperature by cutting off the electric power supply. More than 30 crystals were obtained from this recovered capsule, with crystal sizes between 0.1 and 0.7 mm. The characterization procedures of the recovered crystals were described by Okuchi *et al.* (2015 ▸). A fraction of the crystals was ground and then measured on a powder X-ray diffractometer (Rigaku SmartLab) at room temperature. The lattice constant was *a* = 8.0823 (3) Å, which was significantly larger than that of dry ringwoodite (Sasaki *et al.*, 1982 ▸). By using the previously reported relation between lattice volume and H_2_O concentration (Ye *et al.*, 2012 ▸), we estimated that our recovered ringwoodite crystals contained about 2 wt% of H_2_O. The chemical homogeneity of these crystals was confirmed by measuring the MgO and SiO_2_ concentrations using an electron-probe microanalyzer (Jeol JXA-8800). The homogeneity of hydrogen concentration was confirmed by using a micro-Fourier-transform infrared spectrometer (JASCO FTIR6200-IRT7000). After these successful evaluations, the largest crystal with a volume of 0.14 mm^3^ (0.7 mm × 0.5 mm × 0.4 mm) was selected for single-crystal neutron diffractometry (Fig. 1 ▸).

### 2.2. Neutron time-of-flight single-crystal Laue diffraction   

The crystal was measured using the TOF single-crystal diffractometer TOPAZ at the Spallation Neutron Source, Oak Ridge National Laboratory (Schultz *et al.*, 2014 ▸). The crystal was mounted on a MiTeGen loop using a small amount of Super Glue under a microscope setup. The crystal was measured at 100 K for three and half days using ten sample orientations with 30 Coulombs of proton charge each at the beam power of 1 MW. In our previous study, we have shown that low temperature is effective in decreasing the Debye–Waller factors of ions and increasing the signal-to-background ratio (Purevjav *et al.*, 2016 ▸). The data collection strategy was obtained with *CrystalPlan* software (Zikovsky *et al.*, 2011 ▸), where the completeness of coverage of reflections in reciprocal space was optimized using the orientation matrix obtained from the initial sample orientation. The elliptical integration scheme was used for the observed reflections (Schultz *et al.*, 2014 ▸).

### 2.3. Structure refinement   

**Figure 2:**
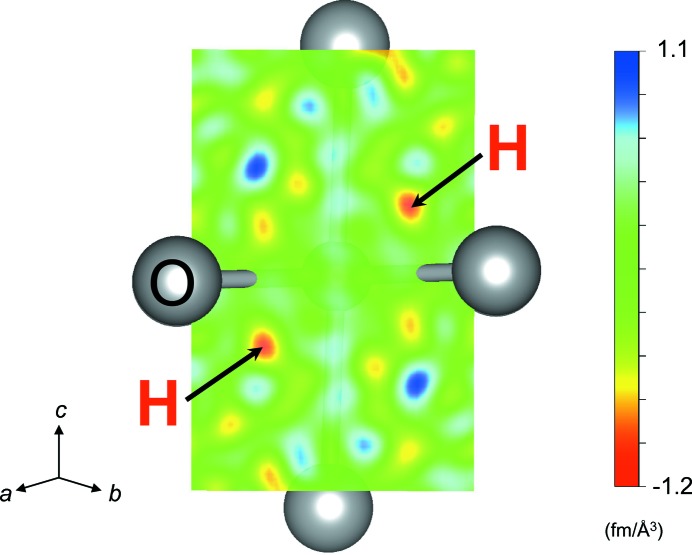
Difference Fourier map showing the negative anomaly of the H^+^ at the *M* site of hydrous ringwoodite. It was obtained from the dataset with *d*_min_ = 0.40 Å and shows the slice on (110).

The obtained neutron diffraction dataset *hkl* was analyzed with the *General Structure Analysis System* (*GSAS*) software (Larson & Von Dreele, 2004 ▸). The observed reflection intensities are proportional to the structure factors, which were Fourier transformed to generate the observed nuclear scattering density distribution in space [*F*_o_(hkl)]. The structure refinement was first performed without H^+^ to obtain tentative structure parameters of Mg^2+^, Si^4+^ and O^2−^. For simplicity, we assumed no disorder between Mg^2+^ and Si^4+^ (Hazen *et al.*, 1993 ▸; Kudoh *et al.*, 2000 ▸). Then, from these parameters we calculated the structure factors, which were also Fourier-transformed into the calculated nuclear density distribution in space [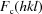]. A difference Fourier map was generated as |*F*_o_| − |*F*_c_|, which is the residual of scattering length density. In this difference map, we found a few negative scattering length anomalies. The most significant anomaly had −1.2 fm Å^−3^ scattering length density in maximum, while the others had −0.9 fm Å^−3^ or smaller density. As mentioned above, H^+^ has a negative scattering length. Hence, the coordinates of each negative anomaly were separately evaluated for determining the actual H^+^ position. H^+^ had a scattering length density of −1.2 fm Å^−3^ at a 192*i* Wyckoff position (Fig. 2 ▸). This significant negative anomaly remained at the same coordinates throughout the refinement procedure. It also showed reasonable O—H bonding distances. The other negative anomalies, in contrast, were unstable in their coordinates and/or have unreliable bond distances (>1.4 or <0.5 Å from the oxygen). Furthermore, refinements including hydrogen on any other negative anomalies all resulted in unreasonably large or negative Debye–Waller factors, so that they cannot be considered as hydrogen.

**Figure 3:**
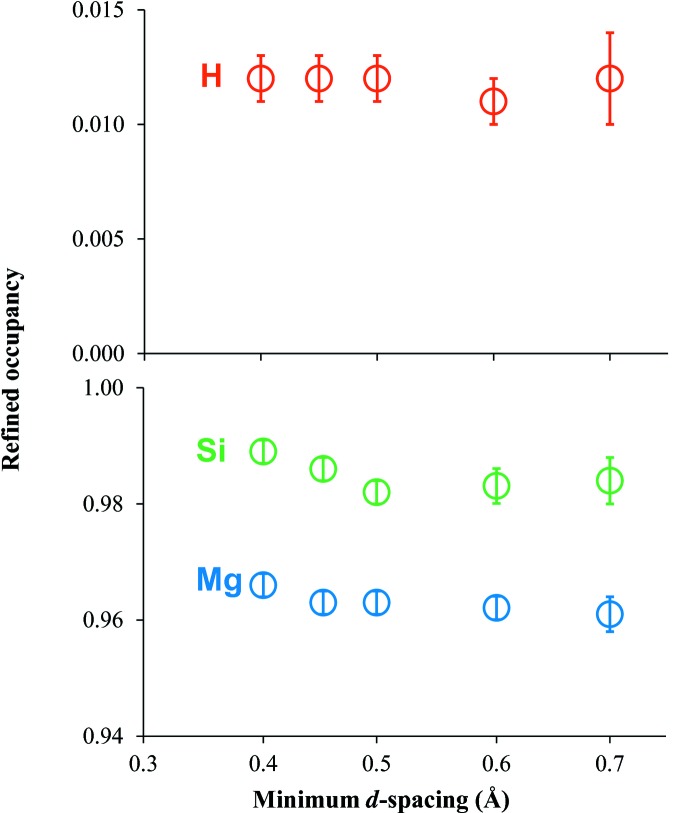
Refined cation occupancies as a function of *d*_min_. Each symbol indicates the result along with error bars of one standard deviation. The numbers of reflections [*I* > 3σ(*I*)] are as follows: 2149 at *d*_min_ = 0.40 Å, 1768 at *d*_min_ = 0.45 Å, 1454 at *d*_min_ = 0.50 Å, 987 at *d*_min_ = 0.60 Å, and 633 at *d*_min_ = 0.70 Å.

In our previous single-crystal neutron diffraction study of hydrous wadsleyite [Mg_1.895_H_0.208_SiO_4_], which has a modified spinel structure, we found that the H^+^ occupancy decreased slightly with increasing *d*_min_ of the refined reflection intensity dataset (Purevjav *et al.*, 2016 ▸). With increasing *d*_min_, the number of reflections was decreased while the uncertainty of H^+^ occupancy was increased. In the current study for ringwoodite, we also performed a series of full-refinement calculations to see the possible variations of cation occupancies at variable *d*_min_ (Fig. 3 ▸). We found that the site occupancy of H^+^ in ringwoodite was stable throughout all these calculations, while those of Mg^2+^ and Si^4+^ were rather stable only at *d*_min_ ≥ 0.50 Å. Thus, it was proved that the refinement result with *d*_min_ = 0.50 Å was the most reliable, providing the highest spatial resolution while keeping the reproducibility of all cation occupancies.

## 3. Results and discussion   

Table 1 ▸ shows crystal data and details of the structure refinement at *d*_min_ = 0.50 Å. Refined structure parameters of each atom at *d*_min_ = 0.50 Å are shown in Table 2 ▸. The H^+^ is located at the 192*i* position which is along the shortest O—O edge of the *M* site. This geometry is qualitatively consistent with our previous powder neutron diffraction study of deuterated ringwoodite (Purevjav *et al.*, 2014 ▸). The O—H bond length of 1.10 (4) Å was 0.20 Å shorter than the O—D bond length. The H⋯O distance was 1.79 (3) Å, which was longer than the D⋯O distance by 0.16 (3) Å. The O—H⋯O angle of 162 (3)° was 9° larger than the O—D⋯O angle. These differences are possibly due to the different numbers of obtained reflections and different spatial resolutions between the powder and single-crystal neutron diffractograms. In the current single-crystal study, the number of reflections is more than 40 times that in our previous powder study. We also enhanced the reciprocal-space resolution in the single crystal compared with the powder study by decreasing *d*_min_ by 0.10 Å. In addition, because of the low-temperature condition of the present study, the statistics were improved to help collecting the reflections of the weaker intensities at the lower *d*-spacings, which was a good contrast to the powder study conducted at room temperature. This obtained O—H distance was nearly comparable to the O—H separation in hydrous wadsleyite determined using single-crystal diffraction [0.999 (5) Å at 100 K] (Purevjav *et al.*, 2016 ▸), and it was also consistent with the previously reported first-principles calculation result on hydrous ringwoodite (1.02 Å at 300 K) (Panero, 2010 ▸).

Thus, we conclude that the present O—H length in ringwoodite is much more reliable than that reported in the powder diffraction study. The obtained O—H⋯O angle was smaller by 17 (3)° than the normal hydrogen bonding angle (180°), as a result of the repulsion between neighboring hydrogen cations at the same *M* site (Panero, 2010 ▸). Thus, the hydrogen cations were located on the faces of the *M* site. These bonding geometries are essential for understanding the effects of water dissolution on the chemical and the physical properties of ringwoodite.

**Figure 4:**
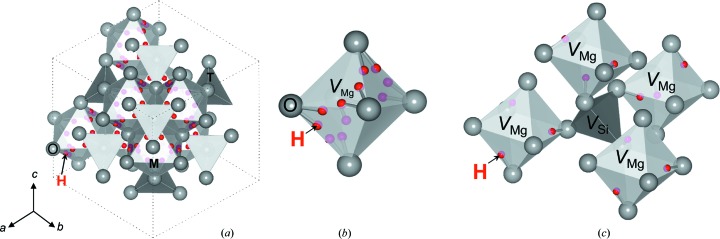
Refined crystal structure of hydrous ringwoodite (*a*) along [111] and (*b*) hydrogen sites in a vacant *M* site. The most plausible model (3H^+^ at 192*i* sites within an Mg-vacant octahedron) is also shown in (*c*). These crystallographic illustrations were created using the *VESTA*3 (Momma & Izumi, 2011 ▸) software.

**Figure 5:**
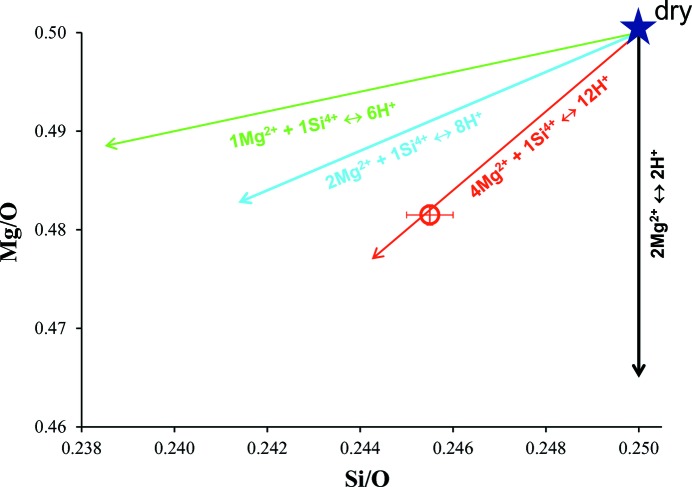
Mg/O and Si/O atomic ratios of the four proposed models (arrows) and the observed result (filled circle).

The refined cation occupancies correspond to the chemical formula of Mg_1.93_H_0.28_Si_0.98_O_4_. The occupancy of H^+^ corresponds to 1.9 (2) wt% of H_2_O, which is consistent with the estimation of about 2 wt% from the lattice constant obtained using powder X-ray diffraction at room temperature. The sum of Mg^2+^ and H^+^ valence charges at the *M* site was 2.07. This value was definitely larger than the stoichiometric value of 2.00, indicating that there was real excess valence charge of H^+^ at the *M* site. There are 12 equivalent positions within each of the Mg-vacant octahedron for H^+^ cations located at the 192*i* sites (Fig. 4 ▸ *b*). However, due to the short distances between each individual position (less than 0.6 Å) only selected positions can be simultaneously occupied. On the other hand, we observed significant deficiency of Si^4+^ occupancy, even though no H^+^ was observed around the *T* site in the difference Fourier map. Combining these results, it is suggested that the vacant *T* site is coupled with the excess valence charge of the *M* site. Then, it is possible to consider four structure models for the distribution of H^+^ in the vacant *M* sites. We calculated the required Mg^2+^ and Si^4+^ occupancies of these four models along with the observed averaged H^+^ occupancy (0.012) based on electrical neutrality constraints (Table 3 ▸ and Fig. 5 ▸).

(1) In the first model, six H^+^ cations are assumed to be in one vacant *M* site, which requires one Mg^2+^ vacancy and one Si^4+^ vacancy (1Mg^2+^ + 1Si^4+^ ↔ 6H^+^). This model requires a larger amount of Si^4+^ vacancy than the observed result.

(2) In the second model, four H^+^ cations are assumed to be in one vacant *M* site, which requires two Mg^2+^ vacancies and one Si^4+^ vacancy (2Mg^2+^ + 1Si^4+^ ↔ 8H^+^). This model requires the same amount of Mg^2+^ and Si^4+^ vacancies, which is also inconsistent with the observed result.

(3) In the third model, three H^+^ cations are assumed to be in one vacant *M* site, which requires four Mg^2+^ vacancies and one Si^4+^ vacancy (4Mg^2+^ + 1Si^4+^ ↔ 12H^+^). The amounts of Mg^2+^ and Si^4+^ vacancies are comparable with the observed values.

(4) In the last model, two H^+^ cations are assumed to be in one vacant *M* site, which does not require any Si deficiency for compensation. Therefore, this model is inconsistent with the observed result.

Thus, we conclude that the third model is the most plausible, where every vacant *M* site involves 3H^+^ (Fig. 4 ▸ *c*).

A previous experimental study demonstrated that the highest H_2_O concentration in ringwoodite was 2.7 wt% (Kohlstedt *et al.*, 1996 ▸). However, the current crystal structure refinement result suggests that this water concentration is not explicitly constrained by the occupancy limitation of the hydrogen site itself. Because there is only one type of oxygen site (32*e*) by crystal space group symmetry, this implies that each oxygen has equal probability to be exchanged by OH^−^, as long as three oxygen atoms out of six in one vacant *M* site are simultaneously exchanged. There is only one crystallographic *M* site in the ringwoodite structure, in stark contrast to the wadsleyite structure having four crystallographic *M* sites and a crystallographically constrained water capacity of 3.3 wt%. It is because only one *M* site type (*M*3) out of the four (*M*1–*M*4) in wadsleyite is available to be exchanged by OH^−^ (Purevjav *et al.*, 2016 ▸). In other words, only one oxygen-site type (O1) out of the four (O1–O4) in wadsleyite is available to be exchanged by OH^−^. Thus, considering the proposed exchange mechanism in ringwoodite, it has a larger water capacity in the lower half of the MTZ in Earth’s mantle, compared with that of wadsleyite in the upper half of the MTZ.

## Supplementary Material

Crystal structure: contains datablock(s) general, I. DOI: 10.1107/S2052520618000616/bm5101sup1.cif

CCDC reference: 1816029

## Figures and Tables

**Table 1:** Crystal data and details of the structure refinement at *d*_min_ = 0.50 Å

Crystal data	
Chemical formula	Mg_1.93_H_0.28_Si_0.98_O_4_
Formula weight	138.73
Crystal system, space group	Cubic, 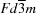
Temperature (K)	100
*a* (Å)	8.0786 (3)
*V* (Å^3^)	527.23 (4)
*Z*	8
	
Refinement	
*wR* (*F*) (%)	4.1
*R* (*F*) (%)	3.4
Reduced χ^2^	2.373
No. of reflections (*I* > 3σ*I*)	1454
No. of parameters	12
ρ (g cm^−3^)	3.495

**Table 2:** Refined structure parameters at *d*_min_ = 0.50 Å The lattice constant is *a* = 8.0786 (3) Å determined by single-crystal neutron diffraction at 100 K.

		Atomic coordinates				
Wyckoff site	Atom	*x*	*y*	*z*	Occupancy	*U*_iso_ † (×10^2^)
16*d*	Mg	0.5	0.5	0.5	0.963 (2)	0.320 (4)
8*a*	Si	0.125	0.125	0.125	0.982 (2)	0.226 (7)
32*e*	O	0.24396 (1)	0.24396 (1)	0.24396 (1)	1	0.325 (4)
192*i*	H	0.353 (4)	0.087 (4)	0.017 (4)	0.012 (1)	4.3 (7)

† Isotropic Debye–Waller factors.

**Table 3:** Calculated and observed Mg^2+^ and Si^4+^ occupancies of the four proposed models These occupancies were calculated based on the observed occupancy of H^+^.

Model	H^+^	Mg^2+^	Si^4+^
6H at *M* site	0.012	0.976	0.953
4H at *M* site	0.012	0.965	0.965
3H at *M *site	0.012	0.953	0.976
2H at *M* site	0.012	0.930	1.000
Observed†	0.012 (1)	0.963 (2)	0.982 (2)

† Determined by single-crystal neutron diffraction in the present study.
